# On the Use of the Neutral Sulphites in Diseases of the Stomach

**Published:** 1852-05

**Authors:** W. Jenner

**Affiliations:** London


*On the Uses of the Neutral Sulphites in Diseases of the Stomach connected with the production of Sarcinæ Ventriculi.* By W. Jenner, M. D., London.—A case of the disease described in the last article is made the subject of a clinical lecture by Dr. Jenner. The main symptoms are, copious daily vomiting, flatulence, distention, and the train of symptoms usually referred to malignant disease of the stomach. The yeasty appearance of the ejecta induced the author to examine with the microscope, and he found an immense quantity of *sarcinæ* and *torulæ.* Ordinary treatment failing, he determined to give the sulphite of soda, being led to do so by the known effects of sulphurous acid upon parasitic vegetable growths. The effects were gratifying, as regards relief, but the more ultimate results are not ascertained.

Before taking the medicine the patient vomited daily from 40 to 100 ounces of fluid, loaded with sarcinæ and torulæ: thus on the 1st of April he vomited 100 ounces; on the evening of the 2d, between 70 and 80 ounces, although he had the same morning, from the action of sulphate of zinc, vomited 50 ounces. No vomiting on the 3d. On the 4th, in consequence of the extreme pain and sense of distension, sulphate of zinc was administered.

On the 5th he took half a drachm of the sulphite of potash early in the morning; in the evening he vomited. On the 6th the dose was increased to one drachm. On the 7th he vomited 12 ounces only of acid flnid, on the surface of which there was but a small amount of scum: it contained perfect sarcinæ, but no torulæ.

From the 8th he took three drachms of the sulphite daily, each dose being given in a drachm and a half of water. At six A. M. on the 9th he ejected from the stomach 4 ounces; the sarcinæ were now decidedly less numerous, and there were no torulæ. In the evening of the 6th he again vomited, but the vomited matters were now free from sarcinæ and torulæ, and there was no appearance of fermentation. Between the 9th and 18th he vomited three times, but on neither occasion did the vomited matter contain sarcinæ or torulæ.

On the evening of the 18th the sulphite of potash was omitted; and on the 19th he vomited a fluid in a state of fermentation, containing torulæ, but no sarcinæ. On the 20th he vomited 9 ounces of a similar fluid; these specimens were examined by a most trustworthy observer, Mr. Morris, the physician's assistant. On the 21st there was a thick scum on the surface of the vomited matters, which contained sarcinæ and torulæ in abundance.

The sulphite of potash, in drachm doses, was again administered; no further vomiting occurred till the 27th, when, without any apparent cause, it recommenced; the vomited matters had their old yeast-like odour and appearance, and were loaded with sarcinæ and torulæ. Dr. Jenner now began to suspect that the man could not be taking his medicine regularly; but on inquiry no omission could be discovered. The medicine itself was then tested, and it was found that it gave off no odour of sulphurous acid on the addition of a stronger acid. At the dispensary we learned that a fresh stock of the drug had come in on the 20th. On submitting a specimen to Mr. Graham, he found that it contained no trace of sulphurous acid. At his suggestion, Dr. Jenner prescribed from this time the sulphite of soda, a more stable salt, and one less liable to be decomposed either in the preparation or by keeping.

On the 29th he took half a drachm of the sulphite, which on the 2d of May was increased to a drachm, three times a day. There was no vomiting till the 5th. He now took carbonate of soda, in consequence of a burning pain in the epigastrium; on the 13, 15th, and 16th he again vomited sarcinæ, and the soda was omitted; from this time to the 10th of June the sarcinæ disappeared. During these thirty-three days he ejected 230 ounces of acid fluid. During the former eight days the quantity was 380 ounces.

[*Ranking's Abstract.*

